# Growth performance, carcass, and meat quality traits in broiler chickens reared on plastic-grid flooring, wood shavings, and zeolite-supplemented wood shavings

**DOI:** 10.1007/s11250-024-03915-1

**Published:** 2024-02-02

**Authors:** Mehmet Kaya, Solmaz Karaarslan, H. Değer Oral Toplu, Evrim Dereli Fidan, Mehmet Kenan Türkyılmaz, Ahmet Nazlıgül

**Affiliations:** Department of Animal Science, Faculty of Veterinary Medicine, Aydın Adnan Menderes University, Aydın, 09100 Turkey

**Keywords:** Broiler, Meat quality, Performance, Plastic-grid floor, Wood shavings, Zeolite

## Abstract

This study aimed to assess the growth performance, carcass characteristics, and meat quality traits of broiler chickens raised on plastic-grid floors, traditional wood shavings, and wood shavings enriched with zeolite. The experimental design included the allocation of 504-day-old chicks to three different bedding materials, namely wood shavings, plastic-grid floors, and zeolite litter, each with four replications (42 birds per replicate). The chicks were individually weighed at birth and grouped according to their average body weights. An experiment involving 504 chicks was conducted, with each replicate consisting of 42 male chicks of similar body weight. At the end of the experiment, a total of 120 chickens were slaughtered with 10 chickens selected from each replicate for processing carcass and meat quality traits. The effects of different bedding materials on mortality (*P* = 0.812), body weight (*P* = 0.565), and body weight gain (*P* = 0.569) were not significant. The ANOVA test was conducted to compare the main effects of performance, carcass, and meat quality characteristics. The feed intake was significantly affected in the 2nd, and 3rd weeks (*P* = 0.001; 0.023); in addition, the feed conversion ratio was significant in the 2nd, 4th, and overall period (*P* = 0.003; *P* = 0.026; *P* = 0.038) by the bedding materials. The breast yield (*P* = 0.001), thigh yield (*P* = 0.028), and wing yield (*P* = 0.023) were significant. The type of bedding material used in broiler production significantly influenced the pH_24_ (*P* = 0.030), L* (*P* = 0.037), a* (*P* = 0.000), and CL (*P* = 0.028). It was concluded that both a plastic-grid floor and zeolite supplementation to wood shaving litter did not significantly affect overall growth performance in broiler chickens. However, the plastic-grid floor showed superior effects on breast meat yield and quality characteristics, particularly reducing cooking loss, when compared to zeolite supplementation in wood shaving litter. The plastic-grid floor led to a slight increase in the feed conversion ratio. Consequently, adopting a plastic grid floor emerges as a viable alternative to traditional wood shavings in broiler farming.

## Introduction

In conventional broiler production, three rearing systems are commonly used: litter, cage, and perforated slatted floor systems. The use of bedding materials (such as litter or plastic-grid floor) in poultry rearing is an important factor in improving environmental conditions, as well as the welfare, performance, and meat quality of broilers (Ghanima et al. 2020).

Litter is a material used on the chicken house floor, like wood shavings or straw, to create a comfortable and clean environment for the birds. Litter allows chickens to engage in natural behaviors and helps control moisture and odor. Providing suitable litter is expected to enhance the birds' well-being and performance (Farghly et al. 2018). It was mostly due to these materials' advantageous features, such as their high moisture absorption capacity and ability to provide thermal comfort for birds. However, issues like as limited amounts, high purchase costs, and a lack of viable alternatives have motivated a quest for alternative bedding materials that can maintain ideal performance without sacrificing comfort (Strašifták and Juhás 2023).

Perforated slatted floors can keep chickens away from excreta and maintain good environmental hygiene, therefore reducing the occurrence of disease (El-Maaty et al. 2023). They improve cleanliness and create a drier and healthier environment for birds by allowing manure and waste to pass through. They facilitate waste removal, reduce the accumulation of ammonia and pathogens, and thus, enhance animal welfare. This flooring option is cost-effective, durable, easy to install and clean, and does not require frequent replacement (May et al. 2024). Furthermore, litter is not necessary for perforated slatted floors, and this will reduce the cost and the amount of work. (Wang et al. 2015). However, it does not provide bedding material, which is important for promoting natural behavior and supporting the physiological needs of the animals (Schomburg et al. 2023).

Zeolite is a natural mineral known for its water-absorbing properties. It is used in animal production to manage moisture levels, reduce ammonia emissions, and enhance air quality in poultry houses. By absorbing excess moisture and reducing ammonia levels, zeolite creates a more favorable environment for broilers, potentially improving their well-being and productivity. Various techniques, including the use of zeolite, have been employed to mitigate litter deterioration, leading to better animal welfare (Naseem and King 2018).

The adoption of rearing systems production has the potential to bring significant benefits in terms of broiler health, production efficiency, welfare, environmental sustainability, and economic efficiency. However, the success of these technologies depends on careful implementation, ongoing research, and industry adaptation to these changes. Several studies exist where the effects of different rearing systems such as cages, litter, and plastic-grid floors on boiler chickens (Munir et al. 2019; Ghanima et al. 2020; Soliman and Hassan 2020). However, there is limited information about the effects of using plastic-grid floors and zeolite supplementation to litter on meat quality traits in broiler chickens. Therefore, the present study aimed to investigate the plastic-grid floors, and zeolite supplementation to wood shavings as an alternative to wood shavings litter in terms of performance, carcass, and meat quality traits in broiler chickens.

## Materials and methods

### Animals and experimental design

**Fig. 1:**
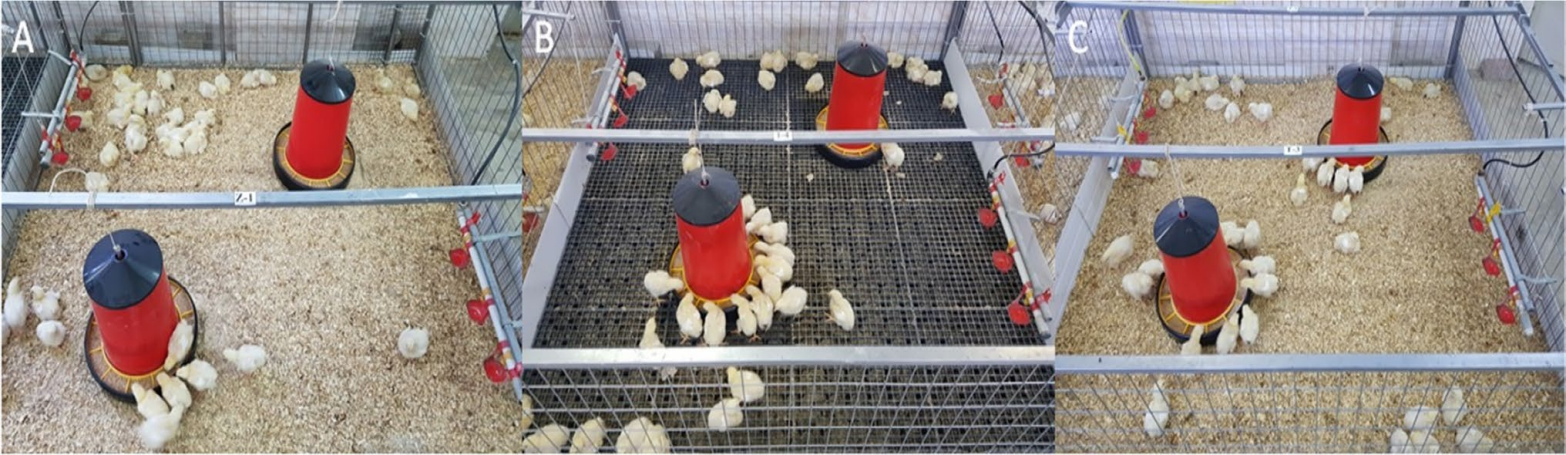
The view of bedding materials, drinkers, and feeders in the pens. **A** 6 kg/m2zeolite + 5 cm layer of pine wood shavings, **B** height: 5 cm; size: 50 × 50 cm; openings: 2 × 2 cm, C: 5 cm layer of wood shavings

A total of 504 one-day-old Ross 308 male broiler chickens were supplied from a local commercial hatchery (EGE-TAV Agriculture and Livestock Investment Trade and Industry Inc., Izmir, Turkey). Marek disease (MD), Gumboro (IBD), and Newcastle disease + Infectious bronchitis (ND + IB) vaccines were injected at day of hatch. Broilers were housed at the Poultry Research Unit (Aydın Adnan Menderes University, Aydın, Türkiye). The separate sex rearing of broilers may be justified if it results in a better growth rate, more efficient utilization of feed, reduced variation that has been designed to more accurately meet the different requirements of each sex, or increased flock uniformity. All broilers were randomly divided into three experimental groups with four replicate pens containing 42 chicks each. Day-old chicks were individually weighed and grouped based on their average body weights. The first group was reared on zeolite bedding material (A: 6 kg/m^2^zeolite + 5 cm layer of pine wood shavings), the second group was provided plastic-grid floor (B: height: 5 cm; size: 50 × 50 cm; openings: 2 × 2 cm), and the third group was designated standard pine wood shavings litter material (C: 5 cm layer of wood shavings) (Fig. 1).

### Housing and general management

Broilers were housed in floor pens measuring 4 m^2^ (2 × 2 m). This area includes the space occupied by two drinkers with six nipples and two round feeders measuring 2 × 0.25 m^2^. The stocking density used in the experiment was 33 kg/m^2^ of broiler chickens. The lighting program followed the guidelines set by the European Union Council Directive (EU 2007). For the first seven days and the last three days before slaughter, a lighting schedule of 23 h of light and 1 h of darkness (23L:1D) was implemented. During the remaining days, the lighting schedule was adjusted to 18 h of light and 6 h of darkness (18L:6D). To maintain optimal conditions for the broiler chickens, the ambient temperature was gradually decreased from 33 °C on day 7 to 21 °C on day 21. It was then kept constant, and the relative humidity was maintained at 40 to 60%. These temperature and humidity levels were recommended in the Ross Broiler's Guide (Aviagen 2018) as suitable for broiler chickens.

The diet (Table 1) was formulated as indicated in the Ross 308 commercial hybrid catalog (Aviagen 2014). In terms of feeding (crumble/pellet diet), the chickens are provided with the diet and water ad libitum, which means they have access to them at all times. The feeders used are of the hanging type, with a feeder length of 2.5 cm per animal.

**Table 1:** Composition of diets during the experiment

Ingredients (g/kg)	Pre-starter (0–10 d)	Starter (11–24 d)	Finisher (25–42 d)
Maize	503	533	568
Soybean meal	398	362	320
Vegetable oil	30.0	40.0	50.0
Wheat bran	27.0	27.0	27.0
Limestone	12.7	12.1	11.3
Dicalcium phosphate	19.2	16.7	14.8
DL-Methionine	2.10	1.70	1.50
L-Lysine	1.30	0.70	0.50
Sodium chloride	3.50	3.50	3.50
Vitamin-mineral premix^1^	2.50	2.50	2.50
*Calculated nutrient composition*			
Metabolizable energy (kcal/kg)	3000	3101	3204
Crude protein, CP	230	215	195
Calcium, Ca	9.60	8.70	7.90
Phosphorus, P	7.60	7.00	6.50
Available phosphorus	4.80	4.30	3.90
Methionine	5.60	5.10	4.70
Lysine	14.4	12.9	11.6

^1^Vitamin-mineral premix contains the following for per kg diet: vitamin A 12.000 IU; vitamin D3 1500 IU; vitamin E 30 mg; vitamin K3 5 mg; vitamin B1 3 mg; vitamin B2 6 mg; vitamin B6 5 mg; vitamin B12 0.03 mg; niacin 40 mg; pantothenic acid 10 mg; folic acid, 0.75 mg; d-biotin, 0.075 mg; choline chloride, 375 mg; manganese, 80 mg; iron, 40 mg; zinc, 60 mg; copper, 5 mg; iodine, 0.4 mg; cobalt, 0.1 mg; selenium, 0.15 mg; and antioxidant, 10 mg

### Growth performance

Each day, the chickens were inspected, and any deceased or removed chicken was recorded for mortality purposes. The body weight (BW) of each broiler was measured individually on a pen and thereafter weekly until 6 weeks of age. The feed intake (FI) was recorded on a pen-weekly basis. Body weight gain (BWG) and feed conversion ratio (FCR) were calculated for each pen. FCR was calculated as the amount of feed consumed per unit of body weight gained. To analyze the growth and feed efficiency of broilers over time, regular estimates of BWG, FI, and FCR were performed for periods of six weeks.

### Carcass and meat quality traits

On 42 d, 120 birds (10 broilers whose body weights were close to the group average were selected from each pen) were selected for processing carcass and meat quality traits. Before slaughtering, the birds underwent a 12-h feed deprivation period and were weighed. The subsequent steps involved slaughtering, scalding, wet plucking, and evisceration of the broilers. After the birds were cut from the neck, they were left for 1–2 min for bleeding. The birds were scalded by placing the carcasses in a 50–60 °C hot water tank for 60–90 s to ensure that the feathers were easily plucked (Banaszak et al. 2021). The wetted bodies were subjected to the first cooling process in cold water after they were plucked in the plucking machine. Later, they were eviscerated, washed, rested in cold water, and drained respectively.

The dressing percentage was determined by dividing the hot carcass weight by the preslaughter weight and multiplying the result by 100. Additionally, the breast, thigh, and wing parts of carcasses were properly separated, weighed, and expressed as percentages of the slaughter weights.

The pH of the breast meat was measured at 15 min postmortem (pH_15_) and 24 h postmortem (pH_24_). The pH measurements were conducted using a digital pH meter (HI 9124 from Hanna Instruments). To assess the meat color, a Minolta CR-400 color meter (Konica Minolta Sensing, Inc. In Osaka, Japan) was used. At the 24-h postmortem, the color measurements were performed on the cranial section of the bony surface of the left filet. The values obtained for L*, a*, and b* represent the lightness, redness, and yellowness of the meat, respectively. The cooking loss (CL) was determined in breast meat samples according to the method described by Honikel (1998) at 24 h postmortem. Briefly, the meat samples placed in plastic bags were cooked in a water bath until an internal temperature of 75 ℃, and then they were chilled for 15 min under running tap water and reweighed. CL is expressed as a percentage and represents the weight loss relative to the initial weight of the breast meat. Water holding capacity (WHC) was evaluated at 24 h postmortem, following the methodology outlined by Grau and Hamm (1953).

### Statistical analysis

The data were statistically performed using the SPSS was used for the analysis (SPSS Version 22.0, IBM Crop., Armonk, NY, US). Using Levene’s test, the assumption of homogeneity of variances was verified. An analysis of variance (ANOVA) was conducted to compare the main effects of performance, carcass, and meat quality characteristics. Significant differences among group means were determined by Duncan's multiple range tests as post-hoc tests. The chi-square test was used for the mortality rate. The level of significance (α) was set at *P* < 0.05.

## Results

In the study, the mortality rate was found to be 2.98%, 2.39%, and 2.98% for wood shavings (WS), plastic-grid floor (PF), and zeolite (Z) groups. The bedding material had no significant influence on the mortality rate (X^2^ = 2.865; *P* = 0.812).

The body weight (BW) of broilers at different growth periods is presented in Table 2. WS, PF, and Z groups had no statistically significant difference in the BW of broilers.

**Table 2:** Body weights of broilers reared on wood shavings, plastic-grid floor, and zeolite supplemented-wood shavings

Time	Bedding Materials			*P*-Value
	WS	PF	Z	
Chick body weight (d1)	47.0 ± 0.11	47.4 ± 0.58	47.1 ± 0.29	0.788
d 7	146 ± 2.89	146 ± 3.43	143 ± 3.31	0.738
d 14	370 ± 3.61	379 ± 8.88	365 ± 5.34	0.340
d 21	784 ± 5.10	809 ± 11.5	779 ± 9.93	0.104
d 28	1325 ± 19.6	1354 ± 45.8	1265 ± 35.3	0.242
d 35	1862 ± 25.7	1888 ± 36.9	1815 ± 38.6	0.404
d 42	2333 ± 54.4	2368 ± 63.8	2271 ± 69.4	0.565

Abbreviations: *WS* Wood shavings, *PF* Plastic-grid floor, *Z* Zeolite

Body weight gain (BWG) during all experimental periods was not significantly affected by different bedding materials. Broilers in the PF group consumed higher feed (> %13, and > %5 respectively) than other groups during the second (*P* = 0.001), and third period (*P* = 0.023), and also the overall experimental period (from 0 to 42 days). However, the differences between the groups were not statistically significant. PF group had higher FCR values, compared to the other groups during the second period, whereas, the mean value of this trait was higher in the Z group than in the WS group during the fourth period (Table 3).

**Table 3:** BWG, FI, and FCR at different growth periods in broilers reared on wood shavings, plastic-grid floor, and zeolite-supplemented wood shavings

Time/Traits	Bedding Materials			*P*-Value
	WS	PF	Z	
*d 0–7*				
BWG (g/bird/week)	96.2 ± 3.04	99.1 ± 3.70	99.3 ± 3.42	0.800
FI (g/bird/week)	133 ± 6.59	140 ± 4.51	123 ± 3.28	0.095
FCR (g feed/g gain)	1.34 ± 0.09	1.42 ± 0.03	1.28 ± 0.05	0.382
*d 7–14*				
BWG (g/bird/week)	224 ± 1.20	232 ± 6.02	222 ± 2.69	0.180
FI (g/bird/week)	285 ± 5.87^b^	328 ± 9.73^a^	279 ± 3.03^b^	**0.001**
FCR (g feed/g gain)	1.27 ± 0.03^b^	1.40 ± 0.01^a^	1.25 ± 0.02^b^	**0.003**
*d 14–21*				
BWG (g/bird/week)	413 ± 3.92	428 ± 5.37	414 ± 4.90	0.084
FI (g/bird/week)	568 ± 6.61^ab^	599 ± 10.9^a^	563 ± 5.51^b^	**0.023**
FCR (g feed/g gain)	1.37 ± 0.00	1.39 ± 0.01	1.36 ± 0.02	0.347
*d 21–28*				
BWG (g/bird/week)	540 ± 16.9	546 ± 35.4	485 ± 27.8	0.279
FI (g/bird/week)	662 ± 14.5	732 ± 36.4	673 ± 13.7	0.142
FCR (g feed/g gain)	1.22 ± 0.01^b^	1.34 ± 0.02^ab^	1.39 ± 0.05^a^	**0.026**
*d 28–35*				
BWG (g/bird/week)	536 ± 13.5	527 ± 9.31	553 ± 22.9	0.552
FI (g/bird/week)	1053 ± 31.2	1125 ± 15.7	1046 ± 13.2	0.053
FCR (g feed/g gain)	1.96 ± 0.08	2.13 ± 0.06	1.90 ± 0.07	0.110
*d 35–42*				
BWG (g/bird/week)	470 ± 34.4	486 ± 33.6	456 ± 32.6	0.827
FI (g/bird/week)	1120 ± 52.7	1202 ± 39.4	1149 ± 26.1	0.397
FCR (g feed/g gain)	2.39 ± 0.08	2.49 ± 0.09	2.54 ± 0.13	0.626
*Overall (d 0 to 42)*				
BWG (g/bird/week)	2286 ± 54.5	2321 ± 64.4	2224 ± 69.2	0.569
FI (g/bird/week)	3822 ± 98.2	4129 ± 108	3836 ± 45.1	0.062
FCR (g feed/g gain)	1.67 ± 0.01^b^	1.77 ± 0.01^a^	1.72 ± 0.03^ab^	**0.038**

This means carrying different superscripts within the same row is significantly different (*P* < 0.05)

Abbreviations: *WS* Wood shavings, *PF* Plastic-grid floor, *Z* Zeolite, *BWG* Body weight gain, *FI* Feed intake, *FCR* Feed conversion ratio

Slaughter weight, carcass weight, and carcass yield were not significantly affected by different bedding materials. Breast yield in the PF group was significantly higher than in the Z group (*P* = 0.001). However, the higher values were obtained in terms of thigh (*P* = 0.028) and wing (*P* = 0.023) yield in the Z group, compared with the PF group (Table 4).

**Table 4:** Carcass traits in broilers reared on wood shavings, plastic-grid floor, and zeolite supplemented-wood shavings

Traits	Bedding Materials			*P*-Value
	WS	PF	Z	
Slaughter weight (g)	2348 ± 54.1	2356 ± 48.5	2299 ± 56.8	0.715
Carcass weight (g)	1672 ± 43.1	1653 ± 36.1	1630 ± 44.4	0.772
Carcass yield (%)	71.1 ± 0.26	70.1 ± 0.53	70.7 ± 0.27	0.244
Breast meat yield (%)	13.9 ± 0.15^ab^	14.4 ± 0.17^a^	13.4 ± 0.19^b^	**0.001**
Thigh yield (%)	20.8 ± 0.14^ab^	20.3 ± 0.20^b^	20.8 ± 0.13^a^	**0.028**
Wing yield (%)	4.82 ± 0.04^ab^	4.66 ± 0.04^b^	4.84 ± 0.05^a^	**0.023**

This means carrying different superscripts within the same row is significantly different (*P* < 0.05)

Abbreviations: *WS* Wood shavings, *PF* Plastic-grid floor, *Z* Zeolite

The effects of different bedding materials on the quality characteristics of breast meat are presented in Table 5. The pH_15_ value was not significantly affected by bedding material. However, broilers in the PF group had higher ultimate pH values than the WS group (*P* = 0.030). The lightness (L*) was significantly higher (*P* = 0.037) in the Z group than in the WS group, whereas a* value was lower in the Z group, compared to the other groups (*P* = 0.000). The cooking loss (CL) was lower for the PF group than for the Z group (*P* = 0.028).

**Table 5:** Meat quality traits in broilers reared on wood shavings, plastic-grid floors, and zeolite-supplemented wood shavings

Traits	Bedding Materials			*P*-Value
	WS	PF	Z	
pH_15_	5.87 ± 0.03	5.80 ± 0.03	5.90 ± 0.03	0.161
pH_24_	5.49 ± 0.01^b^	5.55 ± 0.01^a^	5.53 ± 0.01^ab^	**0.030**
L^*^	57.5 ± 0.50^b^	58.4 ± 0.94^ab^	59.8 ± 0.58^a^	**0.037**
a^*^	3.72 ± 0.27^a^	3.14 ± 0.29^a^	2.12 ± 0.20^b^	**0.000**
b^*^	14.1 ± 0.34	13.7 ± 0.35	14.2 ± 0.47	0.632
CL (%)	37.2 ± 0.55^ab^	35.7 ± 0.89^b^	38.2 ± 0.46^a^	**0.028**
WHC (%)	10.4 ± 0.53	11.2 ± 0.59	10.4 ± 0.48	0.503

This means carrying different superscripts within the same row is significantly different (*P* < 0.05)

Abbreviations: *WS* Wood shavings, *PF* Plastic-grid floor, *Z* Zeolite, L* Lightness, a* Redness, b* Yellowness, *CL* Cooking loss, *WHC* Water holding capacity

## Discussion

In this study, the litter material had no significant effect on the growth performance of the broilers, except FCR. BWG and FI from 0 to 42 days of age, and thus final body weight at 42 days of age were not significantly different for broilers reared in WS, PF, and Z groups. However, FCR in the period from 0 to 42 days of age was significantly higher (*P* < 0.05) in the PF group than in the WS group. Similar to this study, Wang et al. (2015) showed that different rearing systems, such as cages, litter floors, and plastic-grid floors, did not influence weight gain and FI in broiler chickens. Heitmann et al. (2020) reported that the flooring system, whether it was a litter floor or a slatted floor, did not have an impact on the body weight gain of broilers. Sunarti et al. (2010) reported that birds raised on litter floors had significantly lower FI and better FCR compared to birds kept on plastic-grid floors. Banaszak et al. (2021) also found that there was no significant difference in BWG until day 10 when the different proportions of zeolite mixture were added to wood shavings litter. However, they observed an increase in BWG from day 1 to 42.

On the contrary, some studies reported different results. For example, Karamanlis et al. (2008) observed greater BWG and FCR with the addition of zeolite to the litter in broilers. On the other hand, Almeida et al. (2017) found that plastic-grid floors resulted in significantly higher BWG compared to wood shavings. It has been reported that plastic-grid floors for broilers to peck at objects can indeed result in higher feed intake. This increased feed intake may contribute to variations in growth performance observed among different rearing systems. In the case of broilers reared in a system that utilizes PF, one potential reason for their increased growth is their lack of direct contact with feces. By minimizing exposure to fecal matter, broilers in PF systems can experience improved overall health, leading to better growth rates. (Wang et al. 2015). In the current study, the broilers in the PF group had numerically higher values for BWG and FI at 0 to 42 days of age, but the differences between the groups were not statistically significant. However, FCR is significantly higher in the PF group than WS group. The differences between the studies could be explained by the differences in housing and management conditions in the studies.

In this study, carcass weight and carcass yield were not significantly different among groups. However, broilers reared in the PF group had significantly higher breast meat yield but lower thigh and wing yield, compared to those in the Z group. The PF group showed lower ability to exercise or walk, which could explain their higher breast meat yield but lower thigh and wing yields. The results regarding carcass traits are partially consistent with a previous study conducted by Wang et al. (2015), who did not observe significant alterations in carcass yield. Schneider et al. (2016) also found that adding natural zeolites to the litter did not affect carcass and cut yields. Topal and Petek (2021) found that broilers raised on partially slatted flooring had heavier carcasses, breast, and wing weights compared to those on fully slatted flooring or littered floors. The litter material can also impact thigh yield by affecting the overall leg health and quality of the broilers. Healthy legs result in better growth and development of thigh muscles, contributing to higher thigh yields. The litter material indirectly affects wing yield by influencing the activity level and movement of the broilers. Some litter materials may encourage more movement and exercise, leading to better muscle development, including the wing muscles (Topal and Petek 2021).

The pH is one of the most important physical meat quality characteristics, which has a direct effect on the other meat quality traits such as color, water holding capacity, cooking loss, juiciness, tenderness, and shelf life (Fletcher 2002). The pH value of meat is the reflection of the amount of muscle glycogen in the pre-slaughter period and also the rate of glycogen conversion into lactic acid after slaughter. (Mir et al. 2017). In the current study, bedding materials significantly affected the ultimate pH value of breast meat. Broilers in the PF group had higher (*P* < 0.05) ultimate pH values than those in the WS group. Similarly, Özbek et al. (2020) found that the pH value of breast meat of broilers reared on plastic-grid floors was higher than that in deep litter housing systems. The influence of bedding materials on the ultimate pH values of breast meat in broilers can be attributed to several factors. Different bedding materials can differ in their moisture-holding capacity, microbial load, and the general environment in the broiler house. These factors can affect the stress level, activity level, and muscle glycogen breakdown rate of broilers and thus influence the ultimate pH values in the breast muscles. In the present study, a possible reason for the higher final pH in the PF group could be the higher breast meat yield of the broilers in this group. A negative correlation between breast muscle weight and muscle glycogen level has been reported in previous studies. The glycolytic potential is lower in heavier breast muscles and this causes a higher ultimate pH value in these muscles (Le Bilhan-Duval et al. 2008; Tasoniero et al. 2016; Dalle Zotte et al. 2017).

Color is an important quality criterion for deboned and skinless raw meat because it is associated with the freshness and quality of meat by consumers (Fletcher 2002; Mir et al. 2017). Meat color traits could be affected by different factors such as genotype, diet, pre-slaughter stress, slaughter, chilling, and processing conditions that influence meat color traits (Mir et al. 2017). In the present study, broilers in the Z group had a higher (*P* < 0.05) L^*^ value, whereas a lower (*P* < 0.001) a^*^ value, than those in the WS group. The redness index value of the breast muscles of broilers in the Z group was also significantly lower (*P* < 0.001) than those in the PF groups. However, no significant difference was recorded between PF and WS groups in terms of meat color traits. These results suggest that broilers in the Z group had a lighter and less red color of the breast muscles than those in the WS group. There is a lack of studies about the effect of plastic-grid flooring and also zeolite-supplemented wood shavings on meat color traits in broilers. Similar to our study, Özbek et al. (2020) found that there was no significant difference between broilers reared on plastic-grid floors and those in deep litter housing systems regarding L^*^, a^*^, and b^*^ values of breast meat. In another study, Banaszak et al. (2021) stated that the addition of zeolite and halloysite at different levels to the wheat straw litter and feed had no significant effect on the color values of breast meat in broilers.

The present study revealed that bedding material significantly affected the cooking loss of breast muscles in broilers. The lower (*P* < 0.05) cooking loss value was observed for broilers reared in the PF group, compared Z group. Breast muscles of broilers in the PF group had also lower cooking loss than those in WS with no statistical difference. This result could be explained by the higher ultimate pH value in this group than in other groups. It is well known that broiler muscles with low pH values have low water-holding capacity, which causes increased cooking loss, drip loss, and decreased tenderness (Barbut 1993; Mir et al. 2017). Furthermore, the lower cooking loss in the PF (preservative-free) group may be linked to a combination of factors, including the protein functionality influenced by the ultimate pH of the meat. Higher ultimate pH values are often associated with better water-holding capacity and reduced cooking loss, as proteins may have enhanced water-binding properties under these conditions. The relationship is complex and can be influenced by various pre-slaughter and post-slaughter factors affecting muscle structure, composition, and biochemical processes. There is no information about the effects of plastic-grid flooring on the cooking loss of meat in broilers. However, Meluzzi et al. (2008) and Avcılar et al. (2019) reported that bedding materials did not affect this trait. The differences among the studies could be attributed to differences in management and environmental conditions in the studies.

## Conclusion

The study findings indicate that neither the utilization of a plastic-grid floor nor zeolite supplementation to wood shaving litter significantly affected overall growth performance in broiler chickens. However, the plastic-grid floor showed superior effects on breast meat yield and quality characteristics, particularly reducing cooking loss, when compared to zeolite supplementation in wood shaving litter. It is noteworthy that the plastic-grid floor led to a slight increase in the feed conversion ratio. Consequently, adopting a plastic grid floor emerges as a viable alternative to traditional wood shavings in broiler farming. Nevertheless, a comprehensive evaluation of the litter materials' suitability and potential advantages, including aspects such as hygiene, cleanliness, reduced footpad lesions, improved air quality, ease of inspection, and longevity, is imperative. Further research is required, especially with an emphasis on the economic implications of employing these litter materials in broiler breeding.

## Data Availability

The datasets generated during and/or analyzed during the current study are available from the corresponding author upon reasonable request.
